# Prenatal diagnosis of 1408 foetuses at risk of DMD/BMD by MLPA and Sanger sequencing combined with STR linkage analysis

**DOI:** 10.1186/s12920-023-01746-x

**Published:** 2023-12-01

**Authors:** Chunxiao Hua, Lina Liu, Xiangdong Kong

**Affiliations:** Genetics and Prenatal Diagnosis Center, Department of Obstetrics and Gynaecology, The First Affiliated Hospital of Zhengzhou University, 450052 Zhengzhou, China

**Keywords:** Duchenne muscular dystrophy (DMD), Multiplex ligation-dependent probe amplification (MLPA), STR linkage analysis, Sanger sequencing, Prenatal diagnosis

## Abstract

**Objective:**

This study is a retrospective analysis of the prenatal genetic diagnosis results of 1408 foetuses at high risk of DMD/BMD to provide information for clinical genetic counselling.

**Background:**

Duchenne muscular dystrophy (DMD) is a severe neuromuscular disorder characterized by skeletal and cardiac muscle weakness. With the deepening of disease research, some treatments have been applied in clinics. Therefore, early and accurate prenatal diagnosis can inform pregnancy choices for high-risk families.

**Methods:**

A total of 1316 unrelated DMD/BMD families with confirmed genetic diagnoses were recruited from the Genetic and Prenatal Diagnosis Center of the First Affiliated Hospital of Zhengzhou University. Prenatal diagnosis of 1408 high-risk foetuses was performed by MLPA and Sanger sequencing combined with STR linkage analysis for all families.

**Results:**

Among the 1316 families, large deletions, duplications, and small variants of the *DMD* gene accounted for 70.4% (927/1316), 8.2% (108/1316), and 21.4% (281/1316), respectively. Among 1316 mothers, 863 (65.6%) were carriers, and 453 (34.4%) were not carriers. The rate of *de novo* variants was 34.4% (453/1316) in our study. In addition, gonadal mosaicism was observed in 11 pregnant females. Prenatal diagnosis was provided for 1408 high-risk foetuses; 282 foetuses were identified as male patients, 219 foetuses were female carriers, and the remainder had normal genetics. The results of prenatal diagnosis were consistent with the results of follow-up.

**Conclusions:**

Accurate and rapid prenatal diagnosis can be achieved using MLPA, Sanger sequencing, and STR linkage analysis. Furthermore, germline mosaicism in DMD should not be ignored; considering this, a prenatal diagnosis for all pregnant women with a family history of DMD/BMD regardless of whether they carried disease-causing variants is proposed. Genetic counselling and targeted prenatal diagnosis will continue to be a cornerstone of DMD/BMD family management in the future.

## Background

Duchenne and Becker muscular dystrophies (DMD/BMD) are X-linked recessively inherited neuromuscular disorders resulting from variants in the *DMD* gene (NM_004006.4) and mainly affect males. DMD affects 1 in 3800–6300 liveborn males and is the most prevalent but fatal inherited muscle disease in children [1]. The incidence of DMD is not significantly different across many countries, regions, or races. In China, the prevalence of DMD has been reported at 1:3853 male births, with an estimate of approximately 70,000 patients [2], which is one of the highest numbers among countries. DMD and BMD are rarely identified in females, and many female carriers are asymptomatic but may develop modest cardiac dysfunction [3, 4]. The typical symptoms of DMD are progressive muscular atrophy and myasthenia weakness accompanied by pseudohypertrophy of the gastrocnemius muscle. DMD usually manifests itself in children between the ages of 3 and 5 years, with loss of standing and walking ability before the age of 12 years and death from heart failure or respiratory failure before the age of 20 years [5]. BMD affects 1 in 20,000 live births in males, and its clinical symptoms are milder and tend to progress more slowly than those of DMD. Some people with BMD can live up to 60 years, with heart failure as the leading cause of death [6].

Current treatments, such as glucocorticoids, physical therapy, respiratory therapy, and cardiac management, for DMD focus on improving the quality of life and slowing the progression of symptoms associated with the disease [7, 8]. Emerging therapeutic approaches, including readthrough therapy, exon skipping therapy, vector‑mediated gene replacement therapy, and gene editing therapy, can restore expression of functional dystrophin to treat DMD [9]. These therapies are promising and can lead to a better quality of life for patients with DMD, but they are expensive and of unknown efficacy. Overall, a curative treatment for DMD patients is unlikely to be achieved in the near future. Therefore, genetic counselling and targeted prenatal diagnosis will continue to be a cornerstone of DMD family management.

Two conditions are required to perform a prenatal diagnosis: a definite *DMD* gene variant in the proband and the mother’s carrier status. After obtaining the definite *DMD* gene variant and the mother carrier status in the family, prenatal diagnosis of the foetus can be performed based on the known variant. STR linkage analysis was used to determine the risk haplotype of the foetus, and is an indirect means of prenatal diagnosis, whereas Sanger sequencing or MLPA is a direct means of prenatal diagnosis [10]. In China, MLPA/Sanger sequencing and STR linkage analysis are commonly used to verify each other. In this study, we enrolled 1316 unrelated DMD/BMD families. First, the *DMD* gene variants in the families were analyzed by MLPA, NGS, and Sanger sequencing. Second, the mother carrier status in the families was investigated. Finally, the prenatal diagnosis of 1408 high-risk foetuses was performed using STR linkage analysis and Sanger sequencing or MLPA.

## Subjects and methods

### Study design

**Fig. 1:**
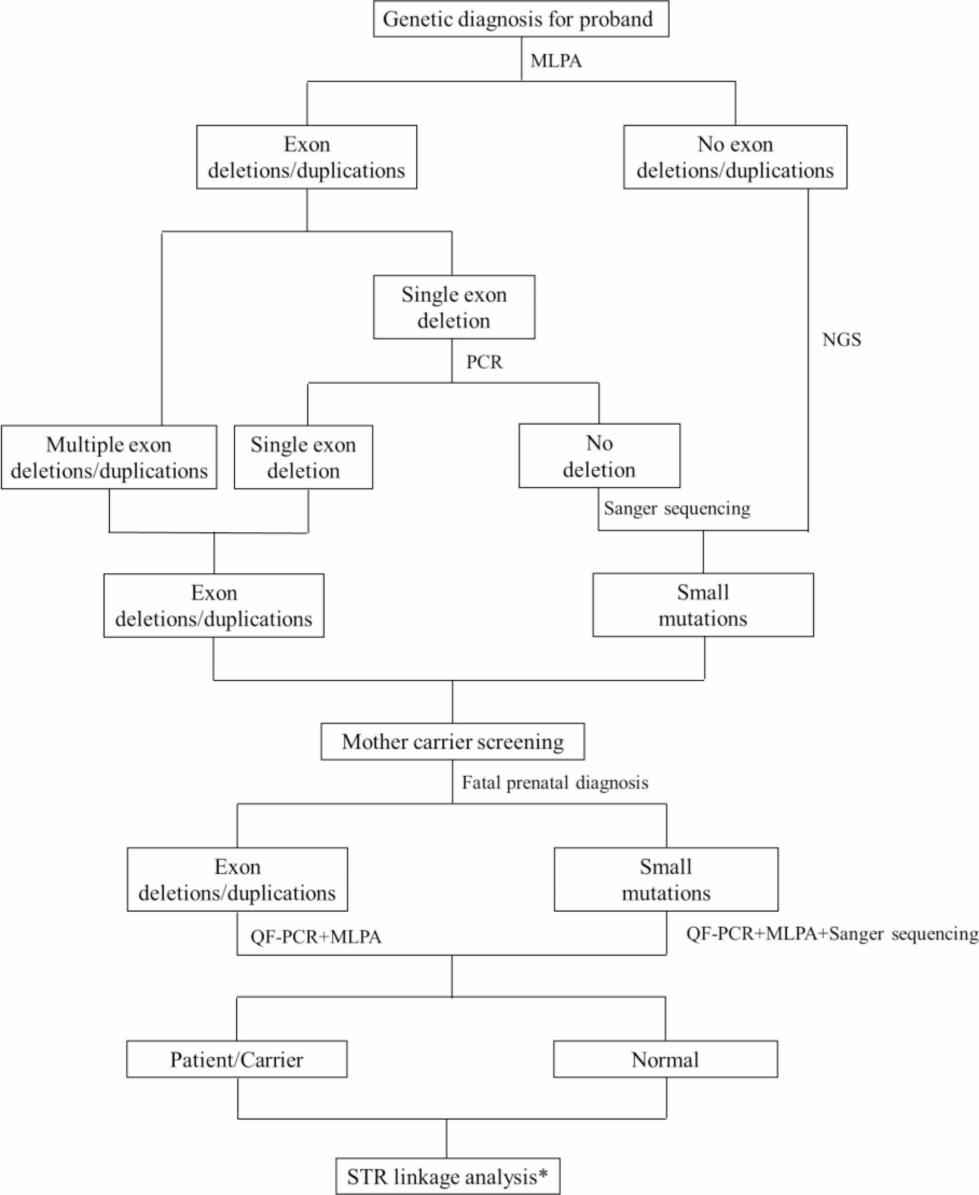
Flowchart for DMD prenatal diagnosis. * STR analysis was performed only when the mother was a carrier

Figure 1 shows the research flow. From January 2005 to February 2022, we recruited 1316 unrelated DMD/BMD families from the Genetic and Prenatal Diagnosis Center of the First Affiliated Hospital of Zhengzhou University. All probands in these families were diagnosed with DMD/BMD based on clinical particular characteristics, increased serum creatine phosphokinase, and molecular genetic analysis. A total of 1316 pregnant women in the families had prenatal diagnoses, and 1408 foetuses were enrolled in our study. All patients signed an informed consent form, as did their families. Furthermore, the First Affiliated Hospital of Zhengzhou University’s Ethics Committee supported this study (2020-KY-0393-002).

## Methods

### Sample collection

Two millilitres of peripheral venous blood was collected from the probands and pregnant females using EDTA-K2 tubes. A DNA purification kit (Tiangen Biotech Company, Beijing, China) was used to extract genomic DNA. Chorionic villus sampling was performed under ultrasound guidance for pregnant women between 10 and 14 weeks of gestation to collect 10–15 mg of chorionic villi. Amniocentesis was performed under ultrasound guidance for pregnant women between 16 and 26 weeks of gestation to collect 10 to 20 ml of amniotic fluid. DNA was extracted from the amniotic fluid or chorionic villus according to the instructions of the DNA extraction kit (Omega Bio-Tek, USA).

### MLPA

Detection of large fragment deletions or duplications of the *DMD* gene using MLPA in all probands, pregnant women and foetuses was performed. MLPA was performed with a SALSA MLPA Kit P034/P035 DMD/Becker (MRC-Holland, Amsterdam, The Netherlands) in accordance with the manufacturer’s instructions. Capillary electrophoresis with an ABI 3130 (Thermo Fisher Scientific, USA) genetic analyser was applied to analyze the amplification products. The MLPA kit’s instructions were followed to determine the copy number, and Coffalyser.net software was used to analyse the original data.

### ***DMD*** NGS panel and data analysis

NGS was performed to screen for small variants in the *DMD* gene in the probands and suspected carriers who were negative by MLPA. To achieve direct and accurate sequencing of all 79 exons and exon/intron junction regions (10 bp), a customized *DMD* gene panel was generated using Ion AmpliSeqTM Designer (www.ampliseq.com). Amplification was performed with the Ion AmpliSeqTM Library kit 2.0, and the template was produced with the Ion PGM™ Template OT2 200 kit and Ion OneTouch instrument. The template was enriched and separated by the Dynabeads MyOne Streptavidin C1 bead kit and Ion OneTouch ES instrument, and sequencing-by-synthesis reactions were carried out with the Ion PGM™ Sequencing 200 kit v2 using the Ion PGM sequencing platform (the sequencing reaction was 500 flows). The sequencing data were processed using Ion Torrent Suite 4.0.2 software. We downloaded VCF files and used Ion Reporter software (https://ionreporter.lifetechnologies.com/ir/) to perform variation annotations. To exclude known polymorphic loci, base mutations, including *DMD* gene indels and substitutions, were screened and compared in the NCBI dbSNP-, Hapmap, and 1000 genome-databases. Missense variants were predicted by PROVEAN, PolyPhen2, and Mutation Taster software, and conservative analysis of amino acids in humans was carried out with the UCSC database. Each of the variants found in this study was searched in Human Gene Mutation Database (HGMD, https://www.hgmd.cf.ac.uk/ac/index.php) and Leiden Open Variation Database (LOVD, http://www.dmd.nl) to identify *de novo* variants.

### PCR amplification and Sanger sequencing

An ABI 3130XL DNA Analyser was used for Sanger sequencing. ABI sequencing Analysis 5.1.1 was applied to evaluate the results and match them to the standard *DMD* reference sequence (GenBank transcript ID: NM_004006.4). A point variant or a small deletion is probably present in the deleted exon if MLPA detects a single exon deletion. Consequently, the exon and its two adjacent exons were amplified by PCR, and healthy people were used as controls. Amplification products were analysed using Sanger sequencing. Sanger sequencing was used to confirm loci with potential pathogenic variants and those with known pathogenic variants identified by NGS.

### STR linkage analysis

Six pairs of fluorescently labelled STR loci for the *DMD* gene were selected, namely, the repeat sequences 5′-(CA)n-3′,3′-MP1P, STR44, STR45, STR49, and STR50. Primers were designed according to standard conditions for STR assays and are presented in Table 1. PCR amplification was performed according to the protocol of Premix Taq™ (TaKaRa). Approximately 2 µL of PCR product,12 µL of Hi-Di formamide, and 0.5 µL of standard were mixed and loaded into capillaries. STR genotypes were obtained by fluorescent capillary electrophoresis with ABI 3130XL Genetic Analyser. Finally, the data were analysed by GeneMapper software v4.0.It should be noted that STR linkage analysis is only performed when the mother is a carrier.

**Table 1:** The sequences of primers used for STR loci

Primer name	Sequence	Length/bp
5′-(CA)n-3′	Forward primer: tcttgatatatagggattatttgtgtttgttatac Reverse primer: attatgaaactataaggaataactcatttagc	206–228
3′-MP1P	Forward primer: atgatcagagtgagtaatcggttgg Reverse primer: atatcgatctagcagcaggaagctgaatg	65–81
STR 44	Forward primer: tccaacattggaaatcacatttcaa Reverse primer: tcatcacaaatagatgtttcacag	180–200
STR 45	Forward primer: gaggctataattctttaactttggc Reverse primer: ctctttccctctttattcatgttac	152–178
STR 49	Forward primer: cgtttaccagctcaaaatctcaac Reverse primer: catatgatacgattcgtgttttgc	223–260
STR 50	Forward primer: aaggttcctccagtaacagatttgg Reverse primer: tatgctacatagtatgtcctcagac	232–244

### Follow-up

After the results of the prenatal diagnosis were clear, we provided detailed genetic counselling to each relevant pregnant woman, including the clinical manifestations of the disease, treatments, and prognosis. The pregnant woman and her family then decide whether to terminate the pregnancy. The first follow-up visit was 1 week after the pregnant woman received genetic counselling to determine whether the pregnancy would be continued. If the pregnancy was to be continued, follow-up occurred at one month after birth, and the creatine kinase level of the newborn was assessed. Genetic testing was performed on the products of conception after voluntary termination.

## Results

### Genetic diagnosis results of probands

**Fig. 2:**
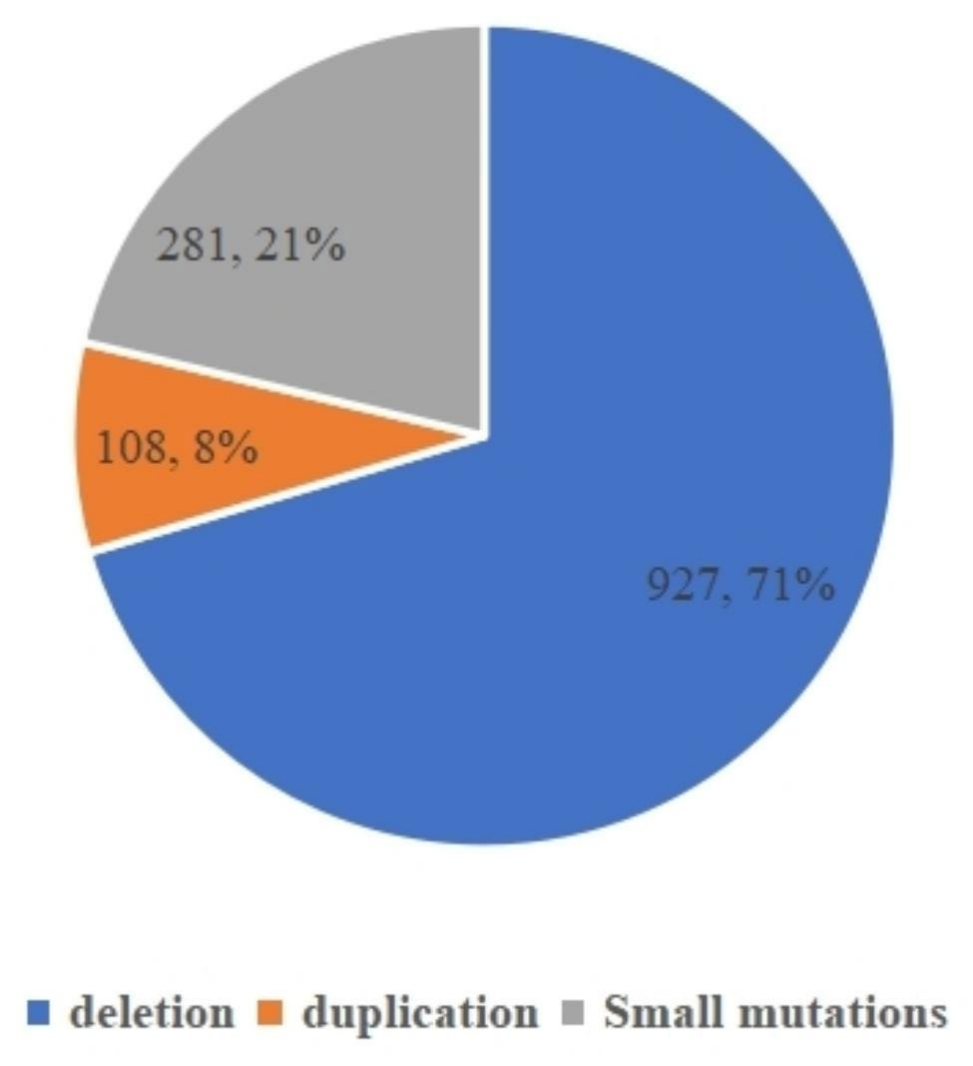
Distribution of *DMD* gene variants in the probands

Among the 1316 DMD families, 927 (70.4%) families had large deletions (≥ 1 exon), 108(8.2%) families had large duplications (≥ 1 exon), and 281 (21.4%) families had small variants. Among these, exon deletions were the most common type of variant, the deletion hotspots were exons 45–50 and the duplication hotspots were exon 2 (Fig. 2). Small variants were distributed in all exons of the *DMD* gene, with no hotspots. In addition, 16 small variants were previously unreported (Table 2).

**Table 2:** New variants found in the study

No.	Types	Location	Variant	Protein
1	Frameshift	9	c.907_908delCA	p.Gln303Glyfs*8
2	Frameshift	24	c.3168delC	p.His1056Glnfs*5
3	Frameshift	28	c.3865delC	p.Thr1291Profs*16
4	Frameshift	34	c.4809_4812dup	p.Ser1605Alafs*2
5	Frameshift	37	c.5156_5157insG	p.Leu1720Phefs*7
6	Frameshift	39	c.5577delT	p.Asn1859Lysfs*6
7	Frameshift	45	c.6567delT	p.Leu2190Cysfs*17
8	Frameshift	51	c.7486_7487delCA	p.Gln2496Glufs*6
9	Frameshift	72	c.10276_10277insGTCCC	p.Pro3426Argfs*21
10	Frameshift	74	c.10490_10494delCCTTA	p.Ser3497fs*
11	Nonsense	35	c.4930 A > T	p.Lys1644*
12	Nonsense	49	c.7170delC	p.Tyr2390*
13	Nonsense	53	c.7789G > T	p.Gly2597*
14	Splice-site	53	c.7660 + 3_7660 + 6delAAGT	————
15	Splice-site	59	c.8925_8925 + 1insACTC TCTCCAAGATCACCT CGAGAAACT CAAG	————
16	Splice-site	69	c.10086_10086 + 1delG	————

### Carrier screening of pregnant females

According to the variant types of the proband, MLPA or Sanger sequencing was used to analyse the carrier status of the mother. The results showed that 863 mothers (65.6%) carried the same variant as the probands, whereas 453 mothers (34.4%) did not (Table 3**)**, suggesting that the proband of the latter may have carried a *de novo* variant or that the mother had a gonadal mosaicism. Among the 453 noncarrier mothers, 11 mothers later gave birth to a newborn with DMD or a carrier with the same *DMD* variant as the proband, and these 11 mothers (2.4%) were considered to have gonadal mosaicism (Table 4**)**. The rate of *de novo* variants was approximately 33.6%.

**Table 3:** Genetic analysis of the *DMD* gene variant

DMD gene variant type	Mother were carrier	Mother were noncarrier	*de novo* variant rate (%)
Exon deletions	535	394	42.1%
Exon duplications	94	15	13.8%
Small variants	234	44	15.8%
Total	863	453	34.4%

**Table 4:** Results of genetic diagnosis in 11 Chimeric families

Family number	Proband	Carrier status of mother	Foetus	Pregnancy outcome
DMD513	Exons 48–52 Deletion	No	Patient	Termination
DMD990	c.8194 A > T(p.Lys2732*)	No	Patient	Termination
DMD2235	Exons 8–34 Deletion	No	Carrier	Delivery
DMD2346	Exons 45–52 Deletion	No	Carrier	Delivery
DMD2416	Exons 45–52 Deletion	No	Patient	Termination
DMD2530	Exons 8–9 Duplication	No	Patient	Termination
DMD2559	Exons 48–52 Duplication	No	Carrier	Delivery
DMD2789	Exons 45–53 Deletion	No	Patient	Termination
DMD2906	Exons 10–11 Deletion	No	Carrier	Delivery
DMD2921	Exons 46–50 Deletion	No	Patient	Termination
DMD3001	Exons 49–52 Deletion	No	Patient	Termination

### Results of prenatal diagnosis

The results of prenatal diagnosis indicated that 282 (20.0%) male foetuses carried the same variant as the proband and would develop DMD/BMD patients in the future, 219 (15.6%) female foetuses were carriers who would pass on their pathogenic variant to the next generation, and the remaining foetuses had no pathogenic variant. The prenatal diagnosis results of 11 noncarrier pregnant women showed that the foetuses carried the same *DMD* variant as the proband, suggesting that these 11 mothers may have had gonadal mosaicism (Table 3). In addition, 21 normal pregnant women (4.5%, 21/463) were at risk of having a foetus carrying the *DMD* gene variant, and 487 carrier pregnant women (51.5%, 487/945) later gave birth to a foetus without *DMD* gene variants.

### Results of STR linkage analysis

In the DMD/BMD prenatal diagnosis for families with a living proband, the combined results of STR linkage analysis and Sanger sequencing or MLPA can provide mutual verification. STR linkage analysis was used to determine the haplotype of the foetus, and the results suggested that the foetus carrying the variant had the risk haplotype as the proband, while a normal foetus did not carry the risk haplotype. The STR analysis results were consistent with those of MLPA or Sanger sequencing.

### Follow-up of pregnancy outcomes

Among all 1408 foetuses, 282 were males expected to develop DMD, and after detailed genetic counseling, the pregnant women chose to terminate the pregnancy. A total of 219 female carriers and 907 healthy foetuses were all delivered at term, and all had normal creatine kinase levels after birth. The results of genetic testing of all products of conception after voluntary pregnancy termination were consistent with those of the prenatal diagnosis.

## Discussion

The standard treatment for treating patients with DMD is corticosteroids and physical therapy [8]. However, this treatment has various limitations, as it is primarily directed towards symptomatic relief and does not alter the ultimate outcome of the disease. Gene therapy, including readthrough therapy, exon skipping therapy, vector‑mediated gene replacement therapy, and gene editing therapy, offers hope for a cure for DMD and aims to restore expression of functional dystrophin [8]. Currently, treatment options for DMD include corticosteroids, such as deflazacort, vamorolone, and prednisone [11], as well as exon-skipping therapeutics approved by the FDA, such as eteplirsen for skipping exon 51 [12], golodirsen and viltolarsen for exon 53 [13, 14], casimersen for skipping exon 45 [15], and the readthrough therapy approved by the EMA, Ataluren [16]. As the effectiveness of therapy depends not only on the therapy itself but also on the stage of the pathological process, early diagnosis of the disease and clinical intervention are very important. Performing prenatal diagnosis can provide sufficient and valuable genetic information that allows DMD families to make appropriate reproductive choices.

In the past, diagnosis of DMD/BMD mainly depended on medical history, clinical manifestations, biochemical examination, muscle biopsy, etc., with a high rate of missed diagnosis and misdiagnosis, and the acceptance of muscle biopsy as an invasive detection method by patients and their families was also low. With the development of molecular diagnostic technology and the deepening of research on *DMD* gene structure, genetic diagnosis is the first choice recommended by guidelines in China and worldwide. After successful cloning of dystrophin in 1987, experts used full-length dystrophin cDNA clones to probe Southern blots, which could directly detect deletions and duplications. The Southern blotting method, which requires high molecular weight DNA and isotopes, is laborious and time-consuming and is now rarely used [17]. After that, hotspots of *DMD* gene deletion were found, and some researchers designed primers for these hotspots; this could effectively detect most deletion variants in DMD/BMD patients but could not detect deletions outside the hotspots, nor could they detect duplication variants and heterozygotes. The *DMD* gene contains microsatellite sequences that are consistent with short tandem repeats and frequently exhibit a high degree of polymorphism in terms of the number of repeats. Because of their different allele lengths, microsatellites are easily detected by PCR. Therefore, prenatal diagnosis in families with a family history can be accomplished by utilizing STR as a genetic marker and PCR amplification for STR linkage analysis [10]. However, the possibility of recombination between microsatellite sequences and unknown variants, the prevalence of sporadic variants, and the lack of information on family members restrict the application of this method. All *DMD* gene exons can be correctly identified by MLPA for deletion/duplication variants, but not all exon regions can be detected for small variants, and there may be false negatives. NGS can detect small variants of the *DMD* gene and has the characteristics of large flux, rapid processing, high accuracy, and a wealth of information provided [18]. Sanger sequencing is more accurate and efficient for the detection of small variants but is expensive and time-consuming to use because the *DMD* gene is large. In the last 18 years, our center has diagnosed thousands of families with a history of DMD and has rich clinical experience in stepwise diagnosis. First, MLPA, NGS, and Sanger sequencing were used to analyse variants in the proband of a family. Second, based on the proband’s variant information, the mother carrier status in the families was investigated. Finally, a prenatal diagnosis was performed on the foetus using STR linkage analysis and Sanger sequencing, or MLPA.

In this study, we recruited 1316 DMD/BMD families, and large deletions, large duplications, and small variants accounted for 70.4% (927/1316), 8.2% (108/1316), and 21.4% (281/1316), respectively. In addition, 16 small variants detected were previously unreported. More than 7,100 *DMD* variants, including exon deletions, exon duplications, and small variants, have been described in the Leiden Open Variation Database (LOVD) and the UMD-DMD France Database. Unlike BMD, which is caused by an in-frame deletion/duplication or a missense variant in the *DMD* gene, DMD is caused by out-of-frame deletions/duplications, nonsense, or frameshift variant [19]. However, it has been found that some patients with nonsense variants or out-of-frame deletions /duplications of specific exons have BMD phenotypes. This is mainly due to the skipping of the exon containing the nonsense variant or causing variable splicing around the deletion exon, resulting in the production of truncated dystrophin and giving rise to the BMD phenotype [20]. Exon skipping has been discovered in some patients with frameshift variants in recent research [21]. Although this situation is very rare, it should be noted. If a diagnosis was made simply based on the classification of the variant, some patients with *de novo* variants may have received an incorrect diagnosis. Therefore, an accurate diagnosis in these cases requires not only genetic testing but also information from the family, dystrophin immunostaining, in silico prediction, and minigene analysis [22].

A total of 863 (65.6%) of the 1316 pregnant females had the same *DMD* gene variants as the proband; the remaining 453 (34.4%) did not. The *de novo* rate was 34.4% (453/1316) in our study, which was similar to previous reports [23]. The *DMD* gene contains 79 exons and has a highly repetitive structure, which is prone to errors during oocyte division; it may be one of the reasons why the *DMD* gene has a high new variant rate [24]. Another reason is that the mother may have gonadal mosaicism [25]. Gonadal mosaicism relates to the presence of a gene variant in some germ cells not present in other tissues of the body. In these cases, the parent with gonadal mosaicism would not show symptoms of the disorder, but they could pass the variant on to some of their children. Grimm et al. [26] found that in DMD patients with *DMD* gene deletion, 58.1% of the patients’ mothers were carriers, and 24.7% of the patients’ mothers were gonadal mosaicism; 17.3% of the cases were *de novo* in meiosis. In this study, 11 foetuses of 463 noncarrier mothers who gave birth again carried the same *DMD* variant as the proband, and it was highly suspected that their mothers had gonadal chimeras. In genetic counselling, the phenomenon of germline mosaicism in DMD must not be overlooked. DMD/BMD is an X-linked recessive disorder in accordance with the law of Mendelian inheritance. Pregnant women who are carriers have a 1/4 chance of giving birth to an affected male patient. Therefore, prenatal diagnosis is advised for pregnant women who had given birth to DMD/BMD patients, regardless of whether they carried the disease-causing variants or not. For DMD/BMD families with a definite diagnosis, it is suggested that other related female family members receive genetic counselling and carrier testing [27].

Our center has been devoted to DMD/BMD research since 2004 and has established a relatively complete platform for DMD genetic diagnosis and prenatal diagnosis. In the early stage, we used STR linkage analysis for prenatal diagnosis, but the intragenic recombination risks cannot be ruled out [28]; thus, STR linkage analysis should not be applied as the only technique in every case of prenatal diagnosis. STR linkage analysis is fast (PCR was amplified approximately 2.5 h, and the capillary gel electrophoretic run took approximately 30 min), accurate, inexpensive, and easy to perform. Furthermore, contamination of foetal sample by maternal blood cells at amniocentesis or chorionic villus sampling can be excluded by performing STR linkage analysis. MLPA or Sanger sequencing can provide direct prenatal diagnosis for the foetus. Based on our experience and the advantages and disadvantages of various technologies, a standardized, efficient, systematic, and economical prenatal diagnosis process has been established in our center. In our center, the basic strategy is to use MLPA, NGS, and Sanger sequencing to identify *DMD* variants in probands, followed by MLPA and Sanger sequencing combined with STR linkage analysis to detect definite variants for a DMD/BMD prenatal diagnosis. Noninvasive prenatal diagnosis of DMD in early pregnancy is also carried out in our center [29]. However, much research is still needed. With the high *de novo* variant rate of the *DMD* gene, whether we should further apply MLPA to screen for exon deletions/duplications in the future on the basis of screening for small variants needs further study. In addition, functional verification is needed for newly discovered variants to clarify their pathogenicity.

## Conclusions

In conclusion, accurate and rapid prenatal diagnosis can be achieved using STR linkage analysis, MLPA, and Sanger sequencing. Furthermore, germline mosaicism in DMD should not be ignored; considering this, prenatal diagnosis is proposed for all pregnant women with a family history of DMD/BMD regardless of whether they carry disease-causing variants. Genetic counselling and targeted prenatal diagnosis will continue to be a cornerstone of DMD/BMD family management in the future.

## Data Availability

The datasets generated and/or analysed during the current study are not publicly available because the file includes a large number of high-throughput sequencing data that are too large to upload, and it is possible that individual privacy could be compromised after publication. However, they are available from the corresponding author upon reasonable request. The web links of the relevant datasets were as follows: Ion Ampliseq Designer (www.ampliseq.com), Human Gene Mutation Database (https://www.hgmd.cf.ac.uk/ac/index.php), Leiden Open Variation Database ( http://www.dmd.nl), Ion Reporter software (https://ionreporter.lifetechnologies.com/ir/).

## Abbreviations

DMD: Duchenne muscular dystrophy
BMD: Becker muscular dystrophy
MLPA: Multiplex ligation-dependent probe amplification
NGS: Next-generation sequencing
STR: short tandem repeat
